# Macroporous Epoxy-Based Monoliths Functionalized with Anti-CD63 Nanobodies for Effective Isolation of Extracellular Vesicles in Urine

**DOI:** 10.3390/ijms24076131

**Published:** 2023-03-24

**Authors:** Julia Neumair, Claudia D’Ercole, Matteo De March, Martin Elsner, Michael Seidel, Ario de Marco

**Affiliations:** 1 Chair of Analytical Chemistry and Water Chemistry, Technical University of Munich, Lichtenbergstr. 4, 85748 Garching, Germany; 2 Laboratory of Environmental and Life Sciences, University of Nova Gorica, Vipavska Cesta 13, P.O. Box 301, SI-5000 Nova Gorica, Slovenia

**Keywords:** monolith chromatography, nanobodies, extracellular vesicles, affinity purification, CD63

## Abstract

Extracellular vesicles (EVs) have enormous potential for the implementation of liquid biopsy and as effective drug delivery means, but the fulfilment of these expectations requires overcoming at least two bottlenecks relative to their purification, namely the finalization of reliable and affordable protocols for: (i) EV sub-population selective isolation and (ii) the scalability of their production/isolation from complex biological fluids. In this work, we demonstrated that these objectives can be achieved by a conceptually new affinity chromatography platform composed of a macroporous epoxy monolith matrix functionalized with anti-CD63 nanobodies with afflux of samples and buffers regulated through a pump. Such a system successfully captured and released integral EVs from urine samples and showed negligible unspecific binding for circulating proteins. Additionally, size discrimination of eluted EVs was achieved by different elution approaches (competitive versus pH-dependent). The physical characteristics of monolith material and the inexpensive production of recombinant nanobodies make scaling-up the capture unit feasible and affordable. Additionally, the availability of nanobodies for further specific EV biomarkers will allow for the preparation of monolithic affinity filters selective for different EV subclasses.

## 1. Introduction

Extracellular vesicles (EVs) are a class of heterogeneous particles with diameters in the nm range, secreted by any cell type, having a pivotal role in intercellular communication in both physiologic and pathologic processes and detectable in any body fluid [1,2]. Since the analysis of their molecular content possesses a high potential for the diagnosis of diseases [3,4] and EVs of different cellular origin co-exist in biological fluids, an effective protocol for the isolation of sufficient amounts of single EV sub-populations, particularly when rare or highly diluted, would be extremely beneficial for the reliability of diagnostic assessments. The conventional purification methods based on (gel) filtration, gradient ultracentrifugation and asymmetrical flow field-flow fractionation [5,6] are effective and reproducible for the separation of dimension classes but cannot discriminate among EVs of the same diameter that originated from different cell types. However, the biologically and clinically relevant information present inside EVs (proteins, nucleic acids, metabolites) depends on the source cells rather than EV dimension. Consequently, there has been an increasing effort to develop affinity purification methods exploiting ligands selective for EV-sub-type specific antigens and suitable for the immunocapture and separation of EVs of different origin [7,8,9,10,11,12]. Such methods mostly foresee the use of ligand-functionalized affinity substrates, commonly magnetic beads, and result in useful-to-isolate small amounts of vesicles for downstream analyses. In contrast, the implementation of preparative purifications aimed at the selective concentration of EVs from large amounts of complex biological samples to use as biotherapeutic agents or for diagnostics [13] has not been achieved using conventional chromatographic material such as sepharose beads because large components tend to clog columns. This kind of drawback is prevented in chromatographic systems exploiting rigid structures such as monolith columns that can be derivatized with antibodies or binders of different origins to obtain affinity units, usually discs of variable height [14]. Monoliths can be manufactured using alternative components that confer different structural characteristics and provide a variety of active groups suitable for functionalization [14]. Silica and methacrylate monoliths have already been exploited for EV fractionation according to alternative chromatographic principles [15,16,17] and the sequential stacking of single methacrylate monolith elements activated with antibodies specific for different EV biomarkers that have succeeded in fractionating two EV sub-populations [18]. Monolith working capacity is independent of the element dimensions, but the cost of the affinity reagents necessary for its functionalization can rapidly become limiting during volume scaling-up. Therefore, it is meaningful to substitute expensive IgGs with recombinant antibody fragments expressed cost-efficiently in bacteria. Recently, we demonstrated that methacrylate monoliths functionalized with nanobodies effectively purified EVs from different biological samples, but the polymer pores were too small to enable the EVs access to the monolith inner space, and this condition compressed the yields [19]. Therefore, monoliths with larger pores (µm range) are highly appreciated because they can accommodate both capture macromolecules and EVs without impairing the flux of complex samples such as biological fluids. This condition should consequently enhance the yields of purified EVs and enable the processing of large sample volumes without affecting the system pressure because clogging risks are minimized. The epoxy-based monolith used in this work is generated by self-polymerization of polyglycerol-3-glycidyl ether with the Lewis acid boron trifluoride (BF3) as a catalyst, which allows for use in mild temperature conditions. Responsible for the macroporous structure (pore sizes of 22 ± 9 µm and total porosity of 79%) are the characteristics of the soluble porogenic mixture from which the solid polymer will be generated [20]. The polyepoxide groups at the monolith surface are active for further modifications and can be treated with solvents and acids similarly to glass chemistry established for protein microarrays [21]. Both the glass chemistry and the direct conjugation of primary amines to the free epoxides are used for protein/antibody immobilization and to transform the original monolithic filters into affinity purification elements [22].

Most of the work for the optimization of EV purification protocols has been performed using serum and cultured cells [13,23], but also, urine EVs have been the object of specific studies [24,25,26,27] because they represent a valuable and easily accessible resource for non-invasive recovery of diagnostic markers for renal altered functionality [28]. Specifically, EVs participate in kidney development and physiology as well as in both renal regenerative and pathological processes. Recently, a specialist network has provided guidelines to standardize some methodological aspects of urine EV separation [29] to improve the consistency of the recovered data and to minimize the bias introduced by the adopted purification methods [30].

In this work, we describe the successful concentration and recovery of urine EVs by exploiting a conceptually new monolith-based filtration disc functionalized with anti-CD63 nanobodies. Such proof-of-principle application of the new immunoaffinity format indicates the feasibility of the approach, but we expect that the matrix could be functionalized with binders selective for biomarkers specific for EV sub-types to isolate rare, although clinically relevant, EVs from large volumes of different biological fluids.

## 2. Results and Discussion

### 2.1. Monolith Filter Preparation and Characterization

**Figure 1:**
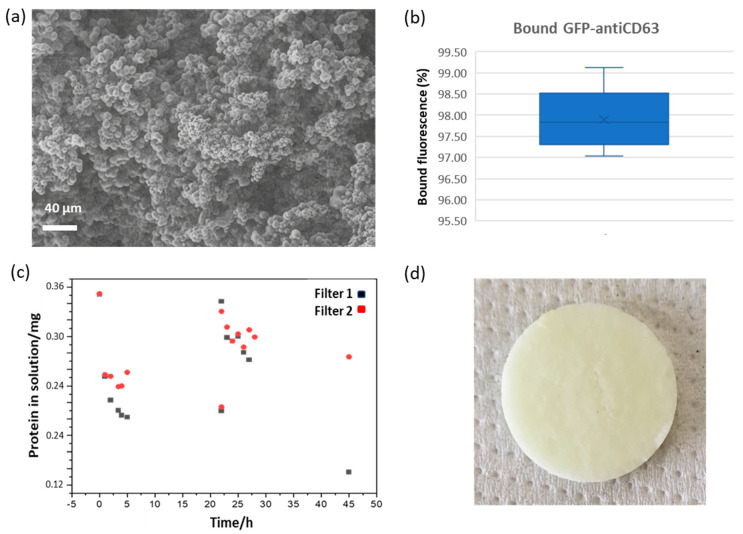
Monolith filter characteristics and its binding features. (**a**) Scanning electron microscope (SEM) imaging of an epoxy monolith disc. (**b**) Binding efficiency of the sandwich between anti-green fluorescent protein (GFP) nanobody and GFP-anti-CD63 nanobody. The data correspond to five independent experiments. (**c**) Evaluation of the binding capacity of the monolith filter. Fluorescent protein was circulated into the column until no apparent further binding was measured. Then, another aliquot of the concentrated solution was added and the protocol repeated. Two independent experiments are reported. (**d**) Macroscopic picture of the filter functionalized with eGFP (note the pale greenish color).

**Figure 2:**
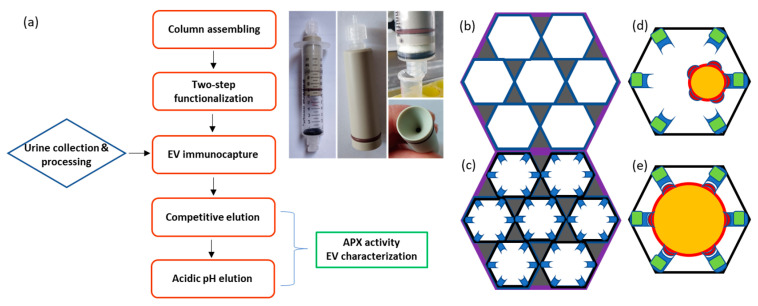
Flowchart of urine EV purification using a monolith filter and its use as chromatographic immunoaffinity matrix. (**a**) The chromatographic column was obtained by inserting the monolith disc into a syringe with hollow piston. Both syringe entrances have an adaptor for plastic tubing that is connected to a peristaltic pump. This helps to control the solution flux through the system. Once the column was assembled, the monolith surfaces were first functionalized with the first protein component, and then, the EV-specific nanobody was bound to it (Supplementary Figure S5). The EVs present in the pre-treated urine sample were selectively immunocaptured and then eluted in two steps, the first competitive and the second obtained by partial nanobody denaturation at low pH. The recovered EVs underwent characterization. (**b**) Schematic representation of a monolith filter structure. The dark areas represent the solid matter composed of globules, the white the internal cavities/pores. (**c**) Disuccinimidyl or epoxide active residues available on the inner matrix surfaces (pores) are used for promoting covalent binding with the primary amines of protein lysines. Filters functionalized with nanobodies specific for EV biomarkers are suitable to capture the EVs present in the mobile fraction (**d**,**e**). Smaller vesicles (**d**) have less surface and probably fewer displayed antigens suitable for binder interactions. The multiple antigens available on larger vesicles (**e**) may induce a stronger avidity effect by enabling the interaction with several binders at the same time.

Monolith filter manufacturing is described in detail in the Materials and Methods section [20]. Pore structure of synthesized monolithic filters was examined using scanning electron microscopy (Figure 1a and Figure S3). The average pore size (22.4 ± 8.8 µm) was estimated analyzing 50 pores from one filter, and this value was in good agreement with the data available in the literature [20,22,31]. The filter globules had an average diameter of 5.7 ± 1.7 µm, as inferred by the analysis of 100 globules (Supplementary Figure S3) and resulted as slightly larger than the value of 4.61 µm described previously [31]. The affinity unit was assembled as depicted in Figure 2a.

### 2.2. Filter Functionalization and Elution Issues

The first attempt to immunocapture EVs from urine samples was performed applying the protocol depicted in Supplementary Figure S4. The monolith filter was functionalized with an anti-CD63 nanobody fused to GFP, and the remaining active residues were blocked with BSA before circulating the urine sample. A sandwich system was prepared to detect the presence of EVs trapped in the column. First, mCherry-fused anti-CD63 nanobodies were biotinylated, flushed over the filter, and then streptavidin–peroxidase was captured by interaction with the available biotin residues. EV actual presence in the column was indirectly confirmed by detecting the conversion of the peroxidase substrate 3,3′,5,5′-tetramethylbenzidine (TMB) over time. However, it was not possible to elute the EVs in glycine buffer, even at pH 2.2, and the addition of detergent (Tween20, 10%) resulted in the recovery of samples negative for the EV membrane biomarker CD63 but enriched in the EV soluble biomarker Alix (Supplementary Figure S4). These results suggest that the harsh elution conditions induced EV lysis rather than promoting their release as integral particles from the functionalized monolith. Difficult EV release from affinity beads and monoliths was often reported [11,15,32], and we reasoned that the strong observed binding between the matrix and EVs could be the consequence of the avidity effect generated by multiple interactions between the nanobody-functionalized monolith pores and the EVs (Figure 2b,c). We also anticipated that these holding force could increase with EV diameter (Figure 2d,e). Such an avidity effect is significantly stronger when nanobodies are used because their small dimension allows for higher density and therefore more binding domains per surface unit that, in the case of soluble antigens, results in higher immunocapture yields with respect to the use of IgGs [33]. Consequently, three alternative capture modules compatible with mild elution protocols were designed (Supplementary Figure S5), and reagents fused to fluorescent proteins were used to simplify the evaluation of the binding efficiency.

In the first option (Supplementary Figure S5, top), a SpyCatcher fused to eGFP was bound to the filter and used to reconstitute a covalent bond with the SpyTag fused to a protease recognition site and the anti-CD63 nanobody. In this case, the nanobody can be cleaved from the anchoring complex by exploiting the 3C protease. In the second case, the anti-CD63 nanobody is fused to an ALFATag and is linked to the matrix through a reversible binding between its tag and a mutant anti-ALFATag nanobody (Supplementary Figure S5, middle). In the third combination, the GFP-antiCD63 nanobody can be released by the filter by inducing at low pH the reversible binding between GFP and the matrix-bound anti-GFP nanobody (Supplementary Figure S5, bottom). Preliminary tests indicated that the third option worked out more efficiently than the others and was selected for the successive protocol optimization steps. The binding and elution conditions were thoroughly analyzed in a set of optimization experiments.

Next, we tried to characterize the modality of protein immobilization on monolithic filter surfaces by direct coupling of the primary amino groups to the epoxy groups using 0.1 M borate, pH 9, as the coupling buffer. EGFP was used as a model protein because the measurement of its fluorescence before and after circulation into the monolithic filter was a convenient method to quantify the signal reduction. When eGFP was directly immobilized on active filter, 42.8% of the loaded protein (0.35 mg) was bound (Supplementary Table S1, left). Successively, the possibility to exploit the immunoaffinity between eGFP and an anti-GFP nanobody to build a pH-dependent bond to monolithic filters was assessed. After eGFP binding to the filter, this was coated with 1% BSA to quench the filter residual active sites before introducing the anti-GFP nanobody. After extensive washing, a visible fraction of the anti-GFP nanobody was recovered in the elution fraction obtained by addition of 0.1 M glycine, pH 2.5 (Supplementary Figure S6, left), confirming that the nanobodies effectively bound to their antigen.

In a second experimental design, the previous set-up was inverted by immobilizing 0.3 mg of the nanobody to the filter, quenching the active sites as above and then circulating the fluorescent protein (0.28 mg). Over time, the fluorescence signal decreased by 55.5% (Supplementary Table S1, middle). In parallel, it was possible to observe that the protein band intensity decreased as well in a sodium dodecyl sulfate–polyacrylamide gel electrophoresis (SDS-PAGE) loaded with samples collected at successive times (Supplementary Figure S6, middle). The addition of acidic buffer determined the eGFP release. Altogether, such results confirmed the effective and reversible binding between nanobody and eGFP.

Finally, a filter was functionalized first with anti-GFP nanobodies (0.72 mg), its active sites were quenched, and the resulting immunoaffinity matrix was used to capture GFP-labeled anti-CD63 nanobodies (0.18 mg). The fluorescence signal was reduced by 97.9% (Supplementary Table S1, right) after incubation, suggesting a very efficient binding of the circulating nanobodies to the captured nanobodies when the latter was in large stochiometric excess. This result was confirmed by SDS-PAGE analysis in which the band corresponding to the GFP-anti-CD63 nanobody nearly disappeared over the incubation time but was recovered in the pH-dependent elution fraction (Supplementary Figure S6, right). This experiment was repeated a further four times using a new monolithic filter and the same amount of proteins every time. The average signal reduction was 97.9 ± 0.8% (*n* = 5, Figure 1b). These results confirmed the high reproducibility of the procedure. Additionally, before starting the purification tests using urine, we wished to determine the maximal amount of protein that can be bound to the monolithic filters and to obtain some information about its binding kinetics. The eGFP solution was circulated over the filter, and its fluorescence was measured every single hour (Figure 1c). The amount of protein in solution decreased, until it reached a plateau after 22 h, and its progressive decrement indirectly suggested its effective capture into the monolithic filter. In two independent experiments, the amounts of immobilized protein per mL of monolithic filter were 0.24 and 0.23 mg. The same filters underwent a second immobilization round that resulted in total amounts of 0.58 and 0.32 mg of bound protein per mL of monolithic filter (Table 1). In parallel, the monolith filter turned its color from white to pale green, indicating that the fluorescent protein removed from the circulating sample was bound to the monolithic filter (Figure 1d). Furthermore, in a control experiment, we did not notice any detectable binding of BSA to the functionalized filter when it was circulated in the system, and this result suggests that unspecific capture of proteins present in the original sample should be negligible.

### 2.3. EV Purification under Optimized Conditions

The adopted protocol (Supplementary Table S2 and Supplementary Figure S7) foresaw a step in which the actual presence of EVs trapped in the filter was indirectly monitored by first loading ascorbate peroxidase (APX) fused to anti-CD63 to the system and then measuring its enzymatic activity (Supplementary Figure S5, bottom). This step can be omitted after protocol optimization but helped to make an interesting observation. It resulted in that the APX-nanobody construct could effectively compete with the matrix-bound anti-CD63 construct and induce the release of some EVs from the filter. These exclusively belonged to the smallest EV fraction (Supplementary Figure S8), and the result confirmed the initial hypothesis that the avidity effect might affect the EV binding proportionally to their size (Figure 2d,e).

**Figure 3:**
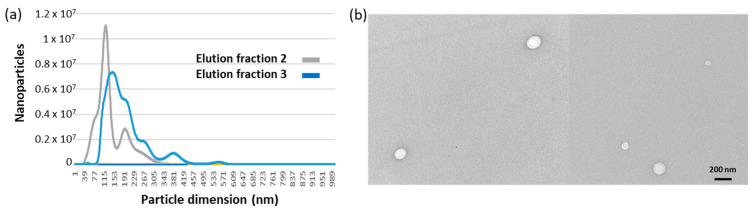
EV size distribution in samples corresponding to different elution fractions. (**a**) Nanosight profiles of immunopurified EVs. EVs bound to the monolith disc functionalized with anti-CD63 nanobodies were eluted by lowering the buffer to pH 2.5. The fraction corresponding to the void volume was removed, and two fractions, each roughly corresponding to the column volume, were collected, and the pH of their buffer was neutralized. (**b**) TEM images of immunopurified EVs.

**Figure 4:**
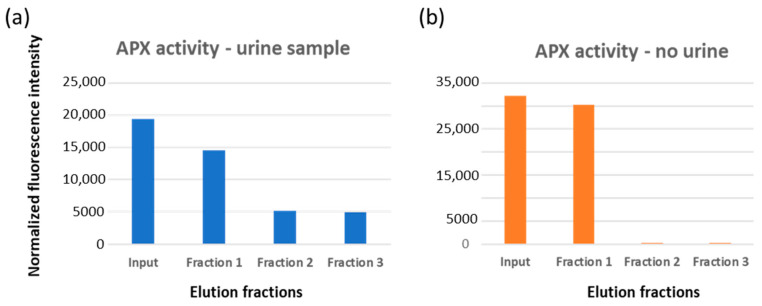
APX peroxidase activity in EV elution fraction. (**a**) The solution containing APX-anti-CD63 (input) was loaded onto a functionalized filter coated with EVs and recovered downstream (fraction 1). After extensive washing, EVs were eluted in 2–3 fractions by addition of acidic buffer. The enzymatic activity of each fraction was estimated by measuring the enzyme substrate conversion into a fluorescent component. (**b**) As a negative control to assess any direct binding of APX-anti-CD63 to the filter, the same experiment was performed in the absence of a urine sample.

The remaining EVs were eluted adding glycine buffer, pH 2.5, into two fractions, whose nanosight profiles are reported in Figure 3a. Particle average diameter for the competitively eluted fraction 1 and the pH-dependent eluted fractions 2 and 3 were 36 ± 4, 126 ± 16, and 139 ± 14 nm, respectively (Table 2). It seems therefore that fraction 1 corresponds to the EV class of exomeres, whereas the others have exosome-like dimensions. It was already reported that immunoaffinity purification preferentially yielded small EVs [25]. The EV integrity, shape and dimension were verified qualitatively by TEM (Figure 3b), APX activity was measured as expected on fraction 1 where the enzyme was probably partly soluble and partly bound to the small EVs. However, a small amount of the APX-anti-CD63 nanobodies resisted to the extensive washing step and co-eluted with fractions 2 and 3 (Figure 4a).

APX did not show unspecific binding for the immunocapture system since it was not held in the filter in a control set. This was performed to functionalize the monolith with the same nanobody sandwich, but only PBS, and no urine, was loaded into the system (Figure 4b). Nanosight analysis also provided an estimation of the amount of purified EVs (Table 2). Altogether, the nanobody-functionalized filter enabled recovery of roughly 3 × 10^10^ EVs from a urine sample of 7.5 mL. The yields are comparable with the best results reported in the literature [24,34,35].

Since the urine volume used for the experiments was chosen arbitrarily, we wished to evaluate whether the filter EV binding capacity was already saturated at these concentrations or might capture more EVs from a larger volume (25 mL); the preliminary results indicated no significant difference in binding capacity. Once the feasibility of the approach is demonstrated in this work, future surveys will more precisely address this issue and further protocol improvements that might require tuning parameters such as the optimal reagent concentrations, the best ratio between the amounts of primary and secondary binders, the incubation times of the different steps, the effect of dynamic versus static incubations and the velocity of flows through the system. Furthermore, the structural characteristics of monolith will allow for simple scaling-up and -down of the platform. Scaling-down would make sense for manufacturing small immunocapture cones to use inside pipette tips (“clinical format”) suitable for concentrating EVs from small biological samples, whereas scaling-up could be applied to recover extremely rare EVs from large volumes, for instance, as could be the case for disease-related EVs in urine or biotherapeutic EVs [13,28]. In this perspective, columns could be manufactured into formats suitable for automated HPLC/FPLC systems, as recently reported for other monolith systems [15,18]. The precise pump control and the on-line quantification of the circulating protein by means of a UV detector will improve the process reproducibility as well as the accurate monitoring of the binding, washing and elution kinetics.

The EV purification experiments reported in this work were performed using an anti-CD63 nanobody [36], namely a binder specific for a generic EV biomarker. In contrast to conventional antibodies, it is easy to produce inexpensively in bacteria, alone or fused to different tags suitable for simplifying directional functionalization procedures and downstream experiments. This approach was very convenient since it allowed for the comparison of several alternative methods during the optimization of the EV purification protocol that required an elevated number of repeats. Furthermore, scaling-up would remain affordable because large amounts of the binders can be purified quickly and cost-effectively. The same principle could be applied in the future for the selection of nanobodies specific for EV sub-populations characterized by the presence of exclusive biomarkers. In this perspective, we already demonstrated the possibility to isolate nanobodies from a phage display pre-immune library panning directly on EVs or on specific epitopes of soluble antigens [8,37].

## 3. Materials and Methods

### 3.1. Chemicals and Proteins

Toluene, tert-butyl methyl ether, trifluoride diethyl etherate, 1,4-dioxane and methanol for the polymerization of the monolithic filters were all purchased from Sigma-Aldrich, subsidiary of Merck (Darmstadt, Germany). Polyglycerol-3-glycidyl ether (CL9) was purchased from Ipox Chemicals (Laupheim, Germany). Nanobodies were subcloned to be produced fused with appropriate tags, purified and analyzed as previously described [8]. The specific anti-CD63 nanobody used for EV capture has a *K*_D_ of 65 nM for its antigen [36]. Streptavidin conjugated to horseradish peroxidase (POX) was purchased from Sigma (18-152, St. Louis, MO, USA). SDS-PAGE gels were stained in Coomassie blue solution for 1 h and destained in 40% methanol and 10% acetic acid. PageRulerTM Prestained protein ladder (Thermo Scientific, Waltham, MA, USA) was used.

### 3.2. Production of Monolithic Filter Discs

The polymerization of epoxy-based monoliths was already published elsewhere [20], and the monolithic filter discs used in this work were produced with adjustments after published protocols [38,39]. In short, polytetrafluoroethylene molds with 16.3 × 60.0 mm of internal diameter were used (Supplementary Figure S1a) to obtain monolithic filter columns that were cut into discs with height of either 3 or 10 mm (Supplementary Figure S1c,d). The porogenic reaction mixture consisting of toluene and tert-butyl methyl ether (60:40, *v*/*v*) was heated to 29 °C. Subsequently, the initiator boron trifluoride diethyl etherate (BF3·Et2O) in 1,4-dioxane (1:10, *v*/*v*) was added to a concentration of 1.25%, and the components were mixed thoroughly. Then, the monomer polyglycerol-3-glycidyl ether (monomer/porogenic mixture ratio 20:80, *v*/*v*) was added, and after vigorous mixing, the solution was filled into the molds and incubated for 45 min at 29 °C (Supplementary Figure S1b). Afterward, the resulting monolithic columns were removed from their molds, stored in methanol overnight and air-dried. Scanning electron microscopy of monolithic filters was performed on a ZEISS SIGMA VP Field Emission Scanning Electron Microscope (Carl Zeiss Microscopy GmbH, Jena, Germany) as described elsewhere in detail [31].

### 3.3. Strategies for Immobilizing Proteins on Monolithic Filter Discs

Two different approaches for protein immobilization on the monolithic filter discs were tested: (i) Jeffamine^®^ ED-2003 (a polyether diamine with dominantly PEG in the backbone) was used as a spacer and *N*-*N*’-Disuccinimidyl carbonate (DSC) for coupling protein primary amines; (ii) direct coupling of proteins via primary amines to the epoxide groups of the monolithic filter discs. For the first approach, monolithic filter discs with a height of 10 mm were submerged in a 2:1 mixture of Jeffamine^®^ ED-2003 and carbonate buffer (15 mM Na_2_CO_3_, 35 mM NaHCO_3_, pH 9.8) and incubated for 48 h at 60 °C. After washing with 60 °C water, discs were activated for four hours at room temperature with a mixture of 93% dimethylformamide, 7% trimethylamine, 49 mM 4-dimethylaminopyridine and 214 mM DSC. After washing with methanol and water, disks were dried and stored at 4 °C until use. The resulting activated monolithic filter discs were first washed with PBS (2 mL/min), and then, the protein diluted in PBS was circulated over night at a flow rate of 0.5 mL/min (Supplementary Figure S2a). For the second approach, monolithic filter discs (height 3 mm) were washed with 0.1 M borate buffer pH 9.0 (2 mL/min), and protein diluted in borate buffer was circulated over night at room temperature at 0.5 mL/min (Supplementary Figure S2b).

### 3.4. Preparation of Monolith Columns and Assessment of Monolith Binding Capacity

The affinity unit was prepared by accommodating a monolith disc into the shell of a 10 mL syringe; an O-Ring (16 × 2.5 mm outer diameter) was placed below the filter and a hollow piston above it. Adapters were inserted on both the piston and the syringe for connecting the filtration unit to tubings (Figure 2a). Discs of different height can be used, and the mobile piston was shifted accordingly. By using a peristaltic pump, the flux of liquids (samples, buffers) through the filter unit can be operated either as a closed system, by connecting the syringe output and input through a tubing, or as an open system, with the input tubing that drives the sample from a tank and the output tubing dropping the content in a waste jar.

Then, the mounted monolith filter was used to evaluate its maximal protein binding capacity by means of circulating fluorescent proteins and monitoring their fluorescence. Filters were equilibrated by circulating 20 mL of 0.1 M borate buffer, pH 9.0, at a speed of 2 mL/min, and then, eGFP diluted in the same buffer was circulated over at 0.5 mL/min. Every hour, circulation was stopped, a sample was taken from the circulation solution, and fluorescence was measured at 485 nm (excitation) and 535 nm (emission) until no further variation was measured (saturation point). After washing in 20 mL PBS, the filter was removed for visive color evaluation.

### 3.5. Preparation of Immunocapture Monolithic Filters

Next, the same mounted monolith filters described above were used to prepare immunocapture systems. A filter (3 mm height) was washed in 20 mL of 0.1 M borate buffer pH 9.0 (2 mL/min) and functionalized by adding 0.72 mg of SpyTag-anti-GFP fusion nanobody resuspended in 2 mL of the same buffer. The protein solution was circulated at 0.5 mL/min over the filter overnight at room temperature. After washing the filter in borate buffer, 5 mL of 1% BSA in 0.1 M borate buffer was circulated for 1 h to block any active sites on the filter surface. The filter was washed with 20 mL of PBS, and then, 800 µL of a 0.23 mg/mL solution of GFP-anti-CD63 fusion nanobody was loaded on the filter and incubated statically for 1 h. Afterward, the filter was washed with 20 mL of PBS. The binding of eGFP-anti-CD63 was assessed by measuring the fluorescence of the sample at 485 nm (excitation) and 535 nm (emission) before and after immobilization. Fluorescence measurements were performed on a Tecan Infinite F200 (Männedorf, Switzerland) and a Perkin Elmer, Viktor X2, 2030 Multilabel Reader (Waltham, MA, USA). As a control, BSA was circulated over the filter functionalized with the anti-CD63 nanobody, and its concentration was monitored reading the absorbance at 280 nm to determine potential unspecific binding to the matrix.

### 3.6. Affinity EV Immunopurification from Urine

First, morning urine was collected and centrifuged at 1800× *g* for 10 min. Until use, samples were stored at 4 °C. For EV isolation, 7.5 (or 25) mL of urine was diluted 1:4 with 20 mM Tris·HCl pH 9.0, and the resulting 30 (or 100) mL of diluted urine was circulated at 0.5 mL/min over the functionalized monolith filters overnight at room temperature. After washing with 20 mL PBS, 800 µL of a 0.24 mg/mL solution of APX fusion nanobody APX-anti-CD63 was incubated statically on the filter for 1 h. Filters were washed again, and EVs were eluted by adding 1.2 mL of 0.1 M glycine buffer, pH 2.5, to the filter. Elution fractions were collected in tubes and immediately neutralized by 1 M borate buffer, pH 9.

### 3.7. Western Blot

For Western blot, proteins separated by SDS-PAGE starting from EV lysates were transferred onto a PVDF membrane using semi-dry transfer at 8 mA for 1.5 h. Transfer buffer consisted of 48 mM Tris base, 39 mM glycine, 1 mM SDS and 20% (*v*/*v*) methanol. Membranes were either directly tested or stored at −20 °C in 5% milk in PBS. Alix was detected with the commercial monoclonal antibody 3A9 (diluted 1:500, Thermo Fisher Scientific, Waltham, MA, USA) and CD63 with rabbit polyclonal antibodies (diluted 1:1000, PA5-92370, Thermo Fisher Scientific) both in combination with HRP-conjugated secondary antibodies.

### 3.8. EV Characterization

The presence of EVs bound to the functionalized monolith filter was assessed indirectly in situ by measuring the horseradish peroxidase-dependent 3,3′,5,5′-tetramethylbenzidine (TMB) color conversion at 450 nm. Specifically, the enzyme was captured by anti-CD63 nanobodies bound to the EV surface that were either biotinylated or fused to a Fc domain. As an alternative, APX activity was measured using the Amplex™ UltraRed Reagent kit (Thermo Fisher Scientific) on the eluted fractions by mixing 100 µL of the sample with 100 µL of the substrate solution [40]. After 15 min of incubation in the dark, fluorescence was detected at λ_Exc_ = 560 nm and at λ_Em_ = 595 nm. EV size was evaluated by Nanosight measurement (NTA version 3.2 Dev Build 3.2.16, Malvern Panalytical Ltd., Malvern, UK) with automatic repeated data acquisition and overall analysis for calculation of particle concentration and dimension. EV morphology was evaluated by TEM: 5 μL of EV suspension was adsorbed on carbon/formvar nickel grids (Electron Microscopy Sciences, Hatfield, PA, USA) and incubated 10 min before removing the sample excess using filter paper. The grids were then coated with 4 μL of staining agent (uranyl acetate diluted 1:3 in distilled water) for further 10 min and washed 5 times in distilled water before being observed in a Philips CM 10 TEM (FEI, Eindhoven, The Netherlands) operated at 80 kV.

## 4. Conclusions

Monolithic affinity chromatography appears as a very effective method for the direct and selective capture and simultaneous concentration of EVs from biological fluids such as urine, because the large pores prevent clogging even after substrate accumulation, and the stable physical structure of the polymeric column can stand an elevated loading rate. These matrix characteristics also assure the scalability of the EV purification system. We demonstrated the approach feasibility using an anti-CD63 nanobody as the immunocapture reagent. However, monolith elements functionalized with binders specific for different biomarkers can be assembled in succession and in combination with further chromatographic elements, exploiting different separation principles such as ion-exchange or size-exclusion and allowing for the simultaneous and selective enrichment of different EV subclasses. The implementation of such a system will require the isolation of suitable binders and, specifically, of small recombinant reagents such as nanobodies that are inexpensive to produce, simple to engineer and are suitable for high-density functionalization of a monolith matrix.

## Figures and Tables

**Table 1:** Characteristics of protein binding to monolith filter.

		Filter 1	Filter 2
First round	µg/mg	0.84	0.85
	mg/mL	0.24	0.23
Second round	µg/mg	1.23	0.34
	mg/mL	0.34	0.09
Total	µg/mg	2.07	1.20
	mg/mL	0.58	0.32

Two rounds of protein binding were performed using two filters. Amount of bound protein is given as µg protein per mg monolithic filter (µg/mg) and as mg protein per mL of monolithic filter (mg/mL).

**Table 2:** Characteristics of the EVs eluted from the monolith filter.

	Particle Number	Particle Average Diameter
Fraction 1 (competitive)	1.13 × 10^10^	36 ± 4
Fraction 2 (pH-dependent)	8.95 × 10^9^	126 ± 16
Fraction 3 (pH-dependent)	9.31 × 10^9^	139 ± 14

Urine EVs immunocaptured from a 7.5 mL sample by anti-CD63 nanobodies bound to the monolith matrix were eluted first by the addition of competitive anti-CD63 nanobodies and successively by decreasing the buffer pH to 2.5. The values are means of three independent measurements performed using samples obtained from different experiments.

## Data Availability

Data will be provided by request.

## Supplementary Materials

The following supporting information can be downloaded at: https://www.mdpi.com/article/10.3390/ijms24076131/s1.
