# Supplementary material for: Macroporous Epoxy-Based Monoliths Functionalized with Anti-CD63 Nanobodies for Effective Isolation of Extracellular Vesicles in Urine
Source: Int J Mol Sci. 2023 Mar 24;24(7):6131. doi: 10.3390/ijms24076131 (PMC10094263; doi:10.3390/ijms24076131)
Supplement: Supplementary file 1 [file ijms-24-06131-s001.zip › ijms-2266414-SI.pdf]

Epoxy-based monoliths functionalized with anti-CD63 nanobodies provide an effective affinity chromatography substrate for extracellular vesicle purification

Supplementary Material

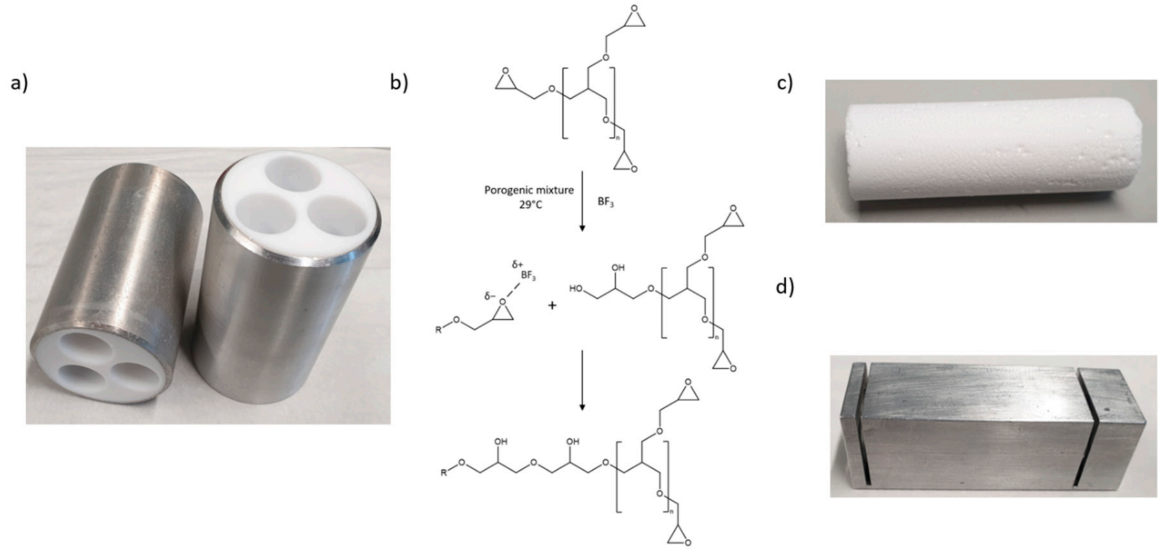

Supplementary Figure S1: Synthesis of monolithic filter discs.

(a) Moulds for synthesis; (b) schematic representation of the polymerization reactions; (c) monolithic filter column after air drying; (d) device for cutting the monolithic filter column into discs with a height of either 3 (left) or 10 mm (right).

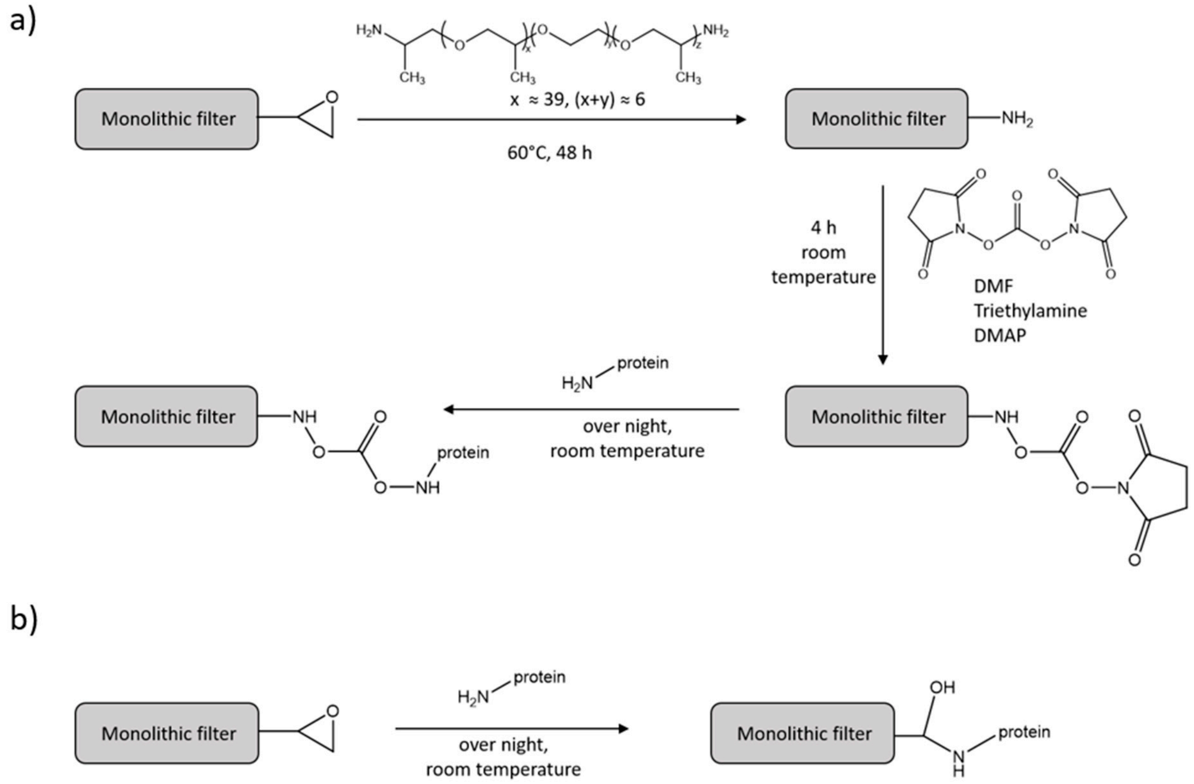

Supplementary Figure S2: Protein immobilization strategies on monolithic filter disc.

(a) The polyether diamine Jeffamine® ED-2003 is bound to the monolithic surface as a spacer and is activated with N-N'-disuccinimidyl carbonate (DSC) in a mixture containing dimethylformamide (DMF), trimethylamine, 4-dimethylaminopyridine (DMAP) before the protein is bound. (b) Proteins bind directly on the active epoxide groups of the monolithic filter.

Pore sizes

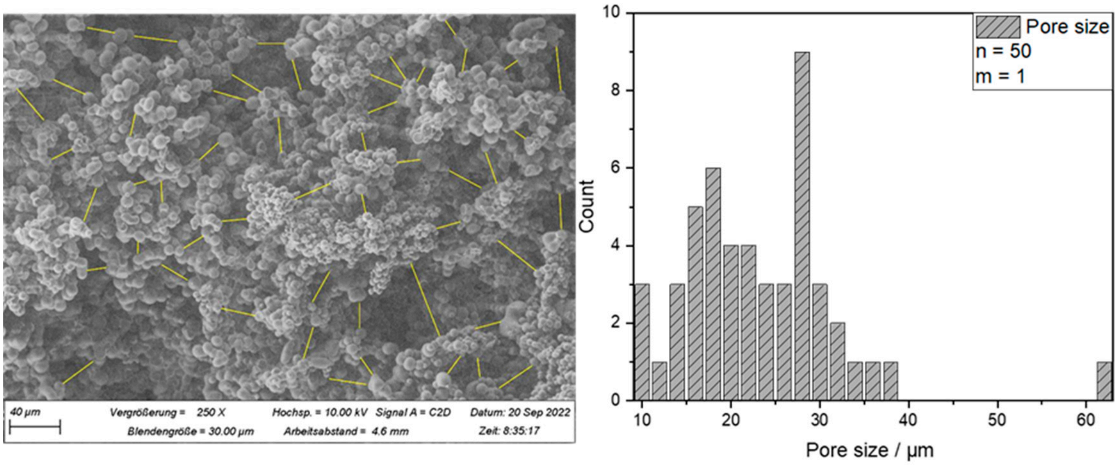

Mean pore size: 22.43± 8.78 µm

Globule diameter

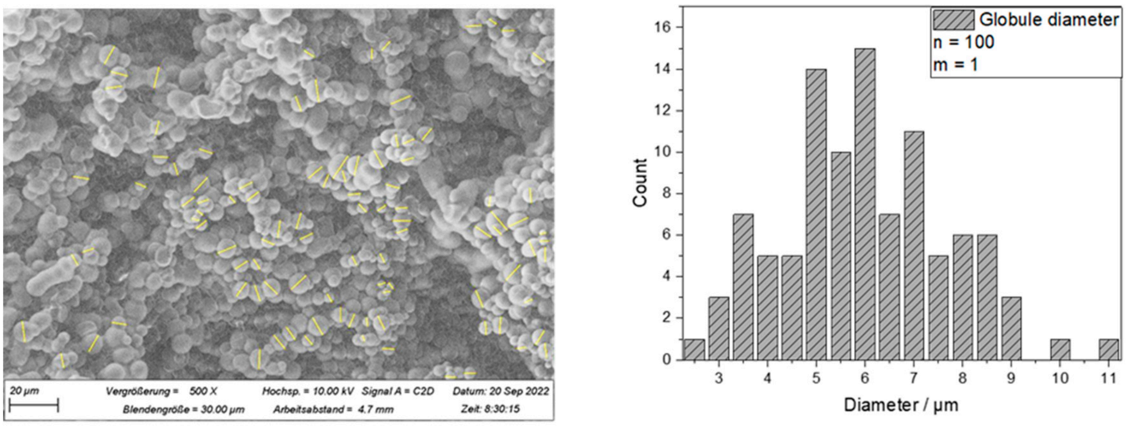

Mean diameter: 5.73±1.69 µm

Supplementary Figure S3. Physical characteristics of the monolith filter.

Monolith filters were analysed by SEM to evaluate the average dimension (yellow bars) and size distribution of both pores (50 measurements) and globules (100 measurements).

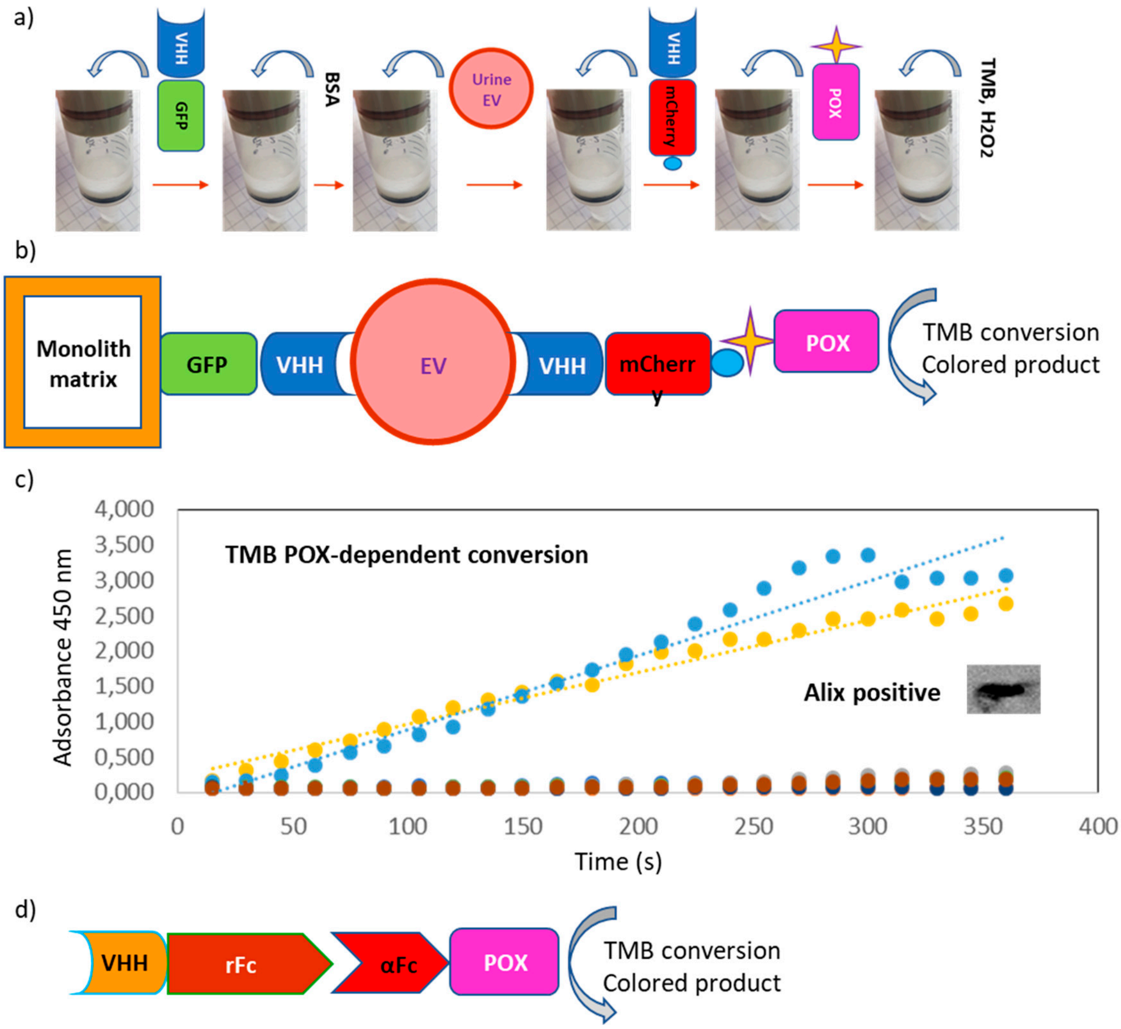

Supplementary Figure S4. EV binding to anti-CD63-functionalized monolith filter.

(a) The EV binding was indirectly detected by measuring the peroxidase-dependent colour reaction. The monolith matrix was first functionalized with anti-CD63 nanobodies (VHH) fused to GFP using diamino-PEG (Jeffamine) and disuccinimidyl carbonate, then the active sites were saturated with BSA, and urine samples were circulated through the filter. Once bound, they served to anchor biotinylated and mCherry-fused anti-CD63 nanobodies. Streptavidin peroxidase was captured on the system, and the enzyme catalysed the conversion of colourless TMB into its oxidized coloured product. (b) Graphical representation of the EV-detection sandwich system. (c) The colour reaction was monitored over the time by means of a continuous flow injection-based immunoassay. Urine was used at different dilution concentrations (blue and yellow signals) and compared with negative controls (brown and grey). The elution of preserved EVs was unsuccessful, but the eluted fraction was positive for Alix in Western blot. (d) An alternative detection system has been tested consisting of an anti-

CD63 nanobody fused to a rabbit Fc domain that could react with a mouse anti-rabbit Fc conjugated to peroxidase.

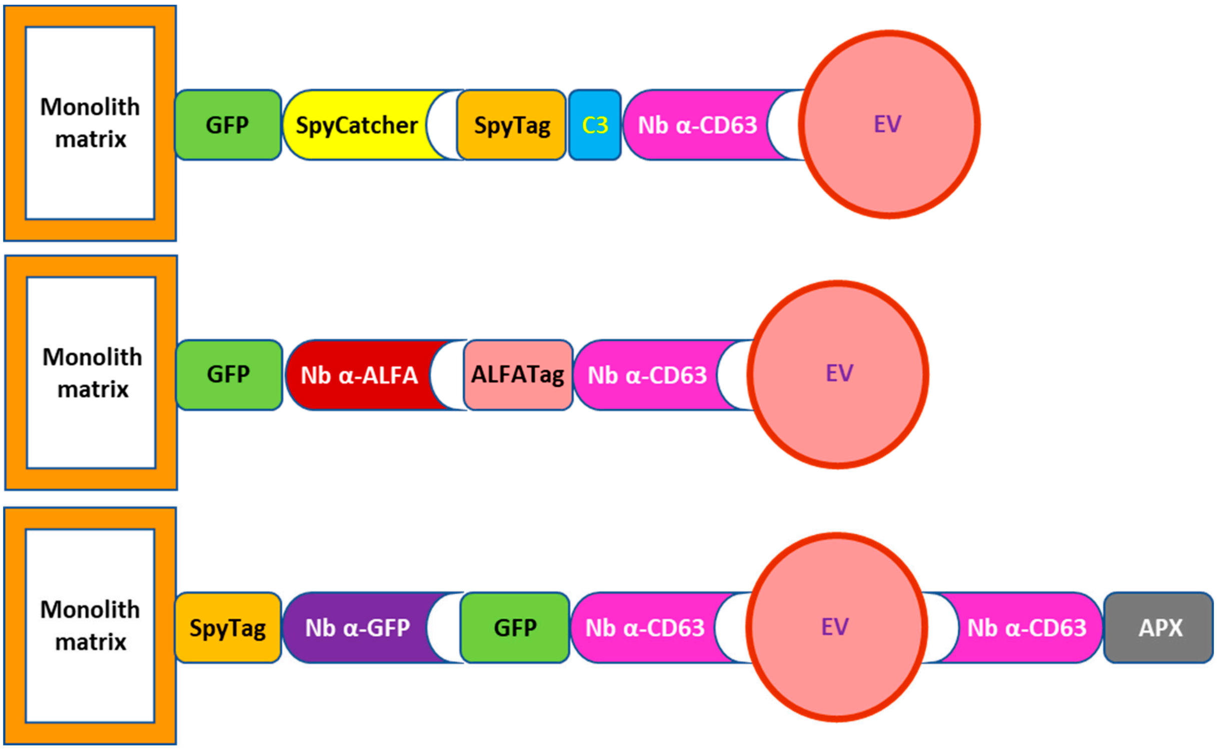

Supplementary Figure S5. Alternative strategies tested for monolith functionalization.

Different combinations have been conceived and tested for obtaining reversible EV binding to the monolith filter, the first based on the possibility to cleave the construct using a C3 protease, the second using the reversible binding between ALFAtag and a mutant anti-ALFA nanobody, and the third using the reversible binding between GFP and anti-GFP nanobody. Immobilized EVs were further bound by a fusion construct formed by the anti-CD63 nanobody and APX that can be used to trigger an APX-dependent colour reaction. The conditions used to activate the monolith with the different protein combinations were the same, as described in Material and Methods for the pair GFP-anti-GFP.

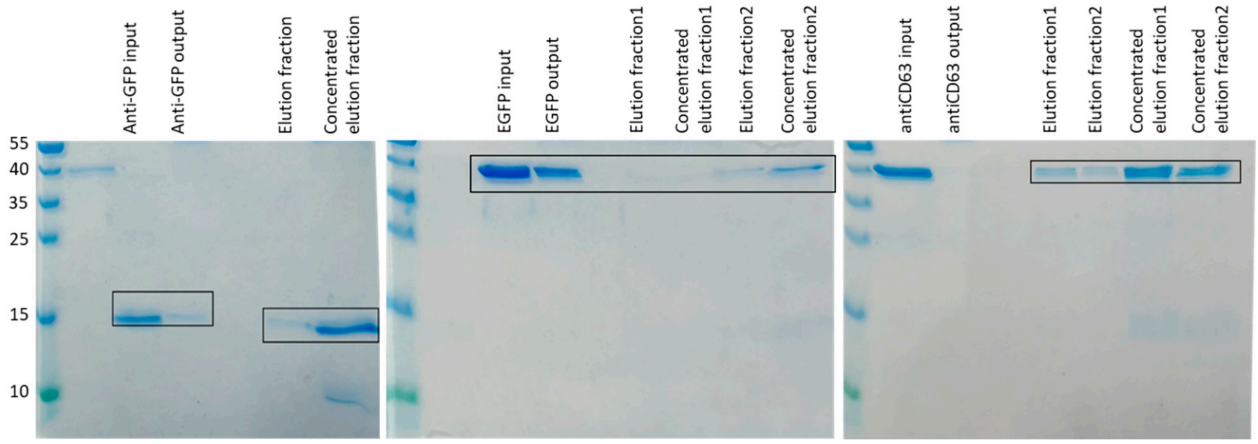

Supplementary Figure S6. Evaluation of the monolith filter immunocapture capacity by SDS-PAGE.

Concentration steps were performed by acetone precipitation, filter quenching using BSA 1% and elution with 0.1 M glycine, pH 2.5. Left: binding of anti-GFP nanobody to a monolithic filter functionalized with eGFP. Middle: binding of eGFP to a monolithic filter functionalized with anti-GFP nanobodies. Right: binding of GFP-anti-CD63 to a monolithic filter functionalized with anti-GFP nanobodies.

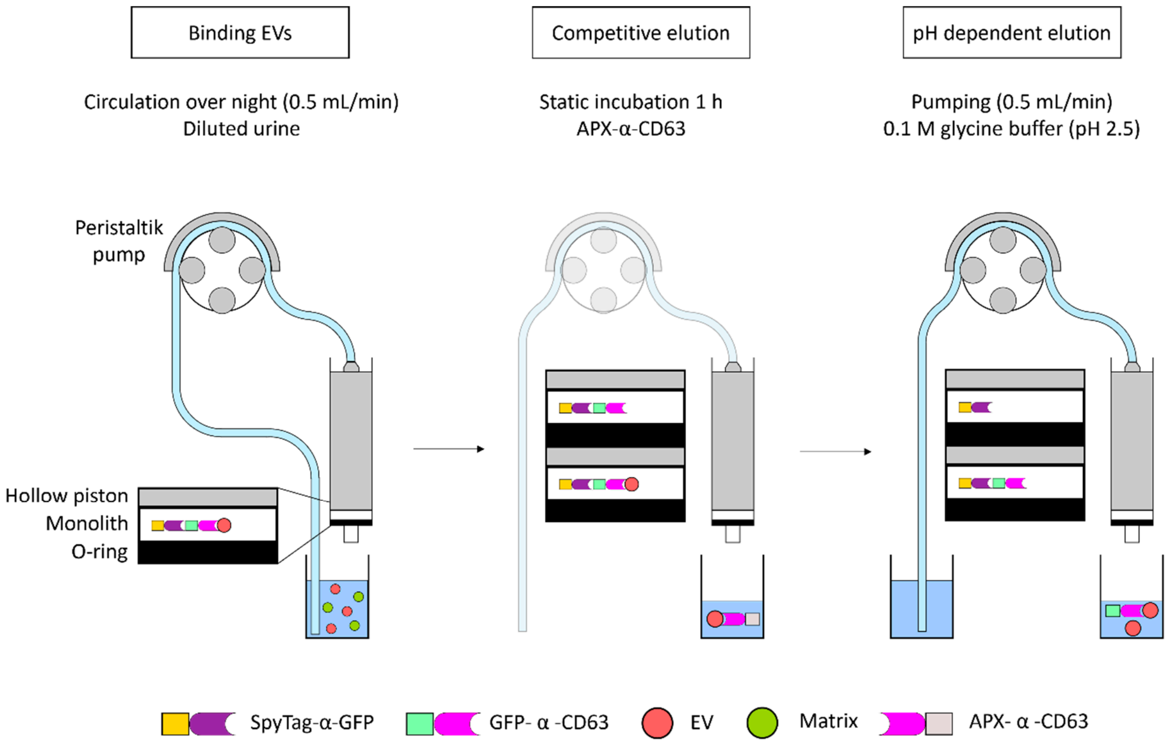

Supplementary Figure S7. Schematic representation of experimental setup.

The characteristics of the experimental setup for each individual step of the affinity EV immunopurification are illustrated. The system was washed after each step using a setup with the same characteristics of that adopted for the pH-dependent elution.

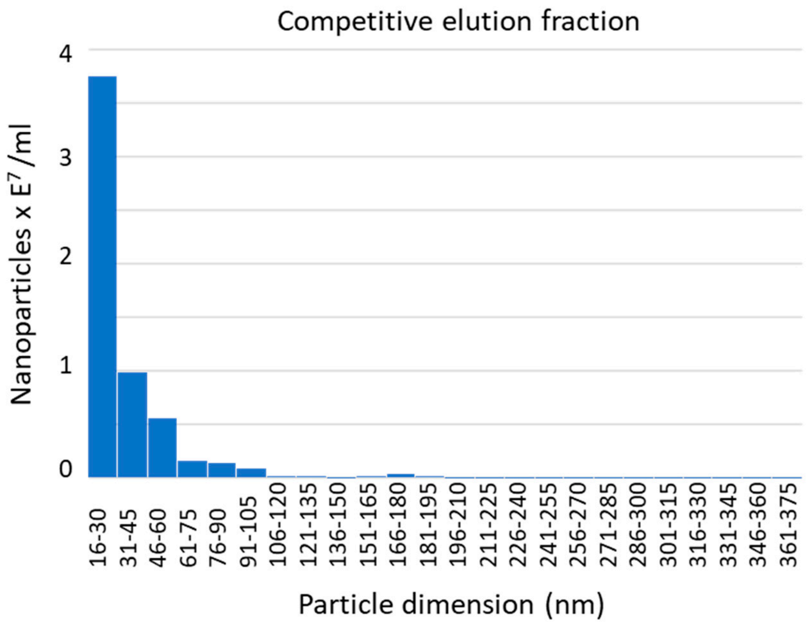

Supplementary Figure S8. Size distribution of the EVs eluted by competition (Fraction 1) according to nanosight measurement.

Supplementary Table S1: Characteristics of protein binding to monolith filters.

Fluorescence was quantified in the sample at the beginning and at the end of the experiment. Left: direct binding of eGFP on active monolithic filter. Middle: eGFP binding to monolithic filter mediated by anti-GFP nanobodies immobilized on the filter and after quenching of residual active sites by BSA coating. Right: GFP-anti-CD63 nanobody binding to monolithic filter after immobilization of anti-GFP nanobodies and quenching of residual active sites by BSA coating.

**Fluorescence**	**On active filter**	**On quenched filter after anti-GFP immobilization**	**On quenched filter after anti-GFP immobilization**
**Initial**	39971	38745	42500
**Final**	22848	17254	890
**Variation**	**42.8%**	**55.5%**	**97.9%**

Supplementary Table S2: Final protocol used for monolith filter functionalization, EV immunocapture and elution.

Anti-GFP = αGFP; anti-CD63 = αCD63

Step	Reagent	Volume / mL	Flow rate / mL/min	Mode	Duration
**Washing**	borate buffer	20	2	flushing	
**Immobilisation**	SpyTag-αGFP	2	0.5	circulation	over night
**Washing**	borate buffer	20	2	flushing	
**Blocking**	BSA	5	0.5	circulation	1 h
**Washing**	PBS	20	2	flushing	
**Immobilisation**	GFP-αCD63	0.8	0.5	static	1 h
**Washing**	PBS	20	2	flushing	
**Immunocapture**	diluted urine	30	0.5	circulation	over night
**Washing**	PBS	20	2	flushing	
**Detection/Elution**	APX-αCD63	0.8	0.5	static	1 h
**Washing**	PBS	20	2	flushing	
**Elution**	glycine buffer	1.2	0.5	flushing	
